# Cytokine-pathway blockers worsen mycosis fungoides masquerading as psoriasis

**DOI:** 10.1016/j.jdcr.2024.04.021

**Published:** 2024-04-24

**Authors:** Gabriela Blanchard, Bettina Bisig, Laurence de Leval, Daniel Hohl, Emmanuella Guenova

**Affiliations:** a Department of Dermatology and Venereology, Lausanne University Hospital (CHUV), Lausanne, Switzerland; b Faculty of Biology and Medicine, University of Lausanne, Lausanne, Switzerland; c Division of Dermatology and Venereology, Geneva University Hospitals, Geneva, Switzerland; d Department of Laboratory Medicine and Pathology, Institute of Pathology, Lausanne University Hospital, Lausanne, Switzerland

**Keywords:** biologics, CTCL, cutaneous: cutaneous lymphoma, folliculotropic mycosis fungoides, JAK-STAT, MF, mycosis fungoides, psoriasiform dermatitis, psoriasiform MF

## Introduction

Mycosis fungoides (MF) is the most common form of cutaneous T-cell lymphoma (CTCL) with a large variety of clinicopathological manifestations. Early-stage MF often resembles more common benign inflammatory dermatoses. Here, we present a case of MF, misdiagnosed as psoriasis for 5 years, and heavily pretreated by multiple cytokine-pathway blockers.

## Case report

**Fig 1:**
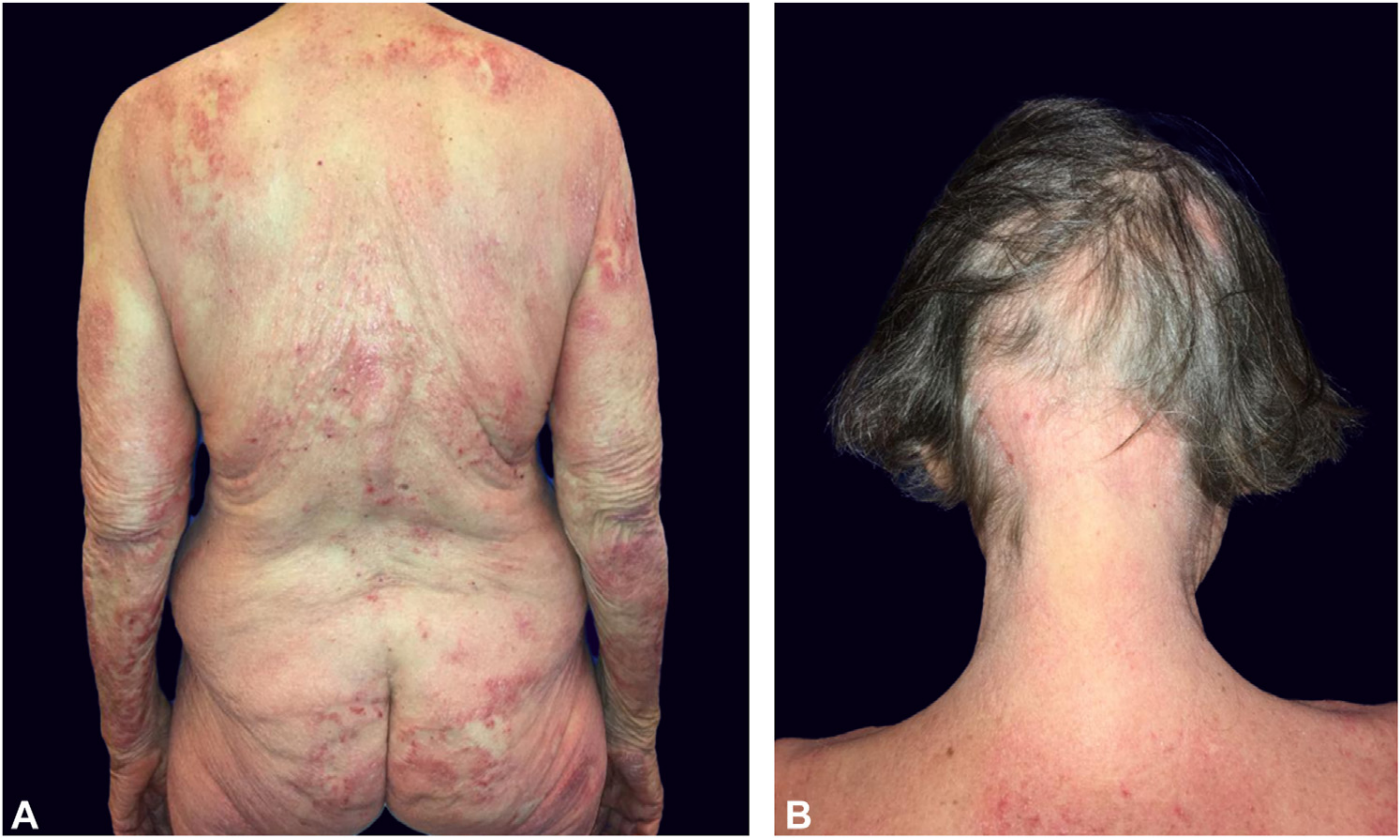
Retrospective clinical images documenting skin manifestations approximately 3 years after disease onset. **A,** Atypical dermatosis, clinically interpreted as psoriasis-like. **B,** Alopecia areata-like patchy hair loss, which had not been considered at the initial diagnostic work-up.

**Fig 2:**
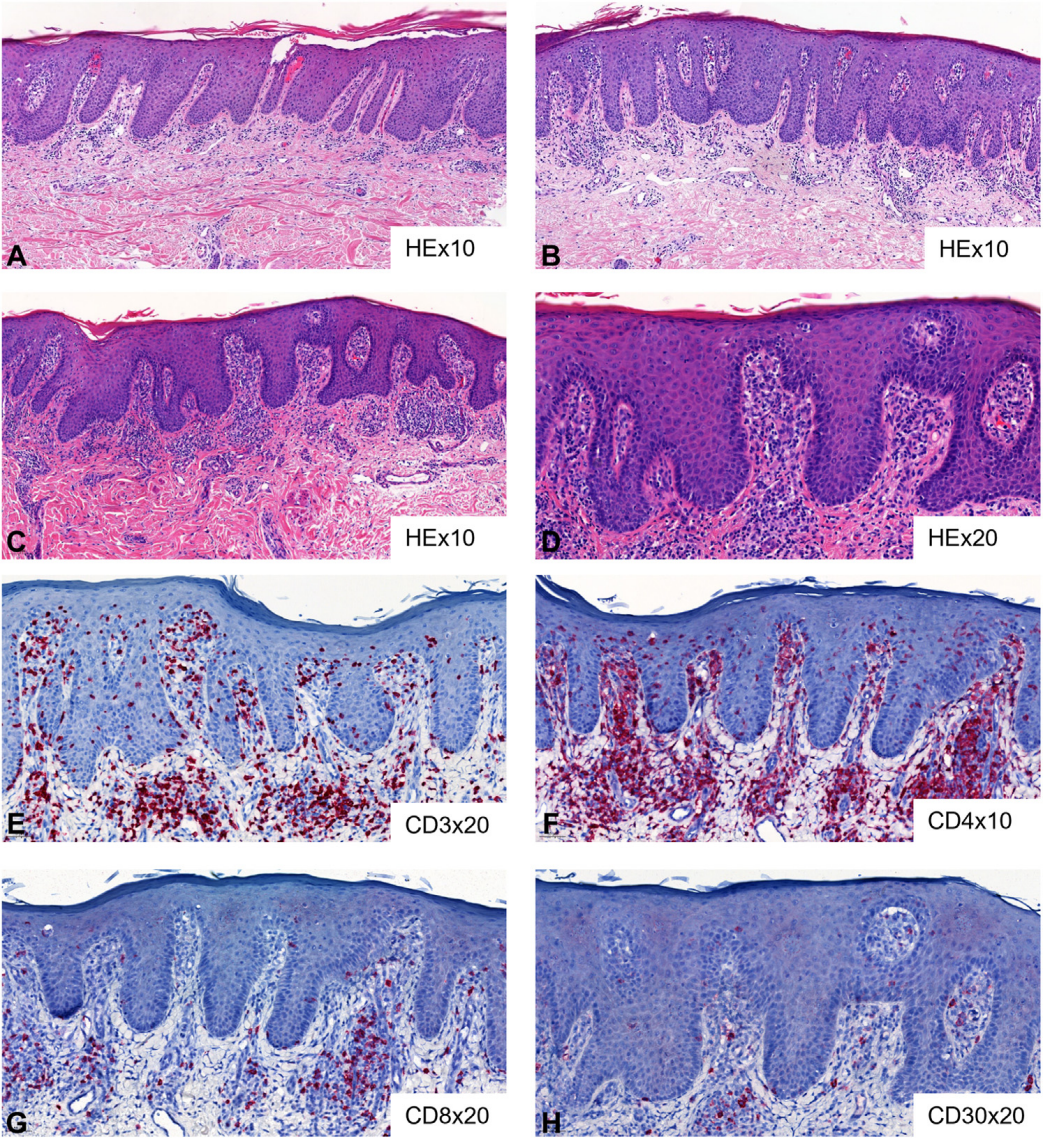
**A** and **B,** Initial skin biopsies showing psoriasiform dermatitis. **C-H,** Skin biopsy after initiation of Janus kinase (JAK)-inhibitor tofacitinib. Biopsy reveals psoriasiform dermatitis with atypical epidermotropic lymphocytic CD4+ infiltrate compatible with mycosis fungoides. *HE*, Hematoxylin and eosin staining.

A 77-year-old female, initially presented at a nonacademic dermatology clinic with numerous well-demarcated, scaly erythematous psoriasiform patches and plaques, as well as multiple disseminated eczematoid lesions (Fig 1, *A*). This comprehensive clinical presentation was deemed atypical. However, 2 separate biopsies had confirmed a histological diagnosis of psoriasis (Fig 2, *A* and *B*), initially leading to the consideration of psoriasis as the primary diagnosis. Based on this assumption, a diagnostic assay was performed to characterize the cytokine profile in the patient’s skin lesions and facilitate a targeted selection for biologic treatment. The assay, performed on 3 independent skin biopsies, revealed a T helper 17 cell (Th17)-skewing in the analyzed skin lesion, further strengthening the assumption of psoriasis. Consequently, systemic treatment with an anti-interleukin-23 (IL23) monoclonal antibody (guselkumab) was introduced but significantly worsened the disease. Subsequent administration of methotrexate had to be discontinued after only 2 months due to side effects, making it challenging to assess its therapeutic efficacy in this particular case. Further, there was no benefit from a 3-month treatment course with the phosphodiesterase 4 inhibitor apremilast. Ixekizumab, anti-IL17A monoclonal antibody, was introduced afterward and maintained for 7 months, during which the patient’s skin continued to deteriorate significantly. At this time, an alopecia areata-like patchy hair loss, which could be appreciated also on retrospective clinical images (Fig 1, *B*), was first documented in the patient’s health records. Treatment with the Janus kinase (JAK)-inhibitor tofacitinib was suggested in the context of a possible association of psoriasis and alopecia areata; nevertheless, 4 months of tofacitinib treatment led rapidly to substantial clinical worsening.

**Fig 3:**
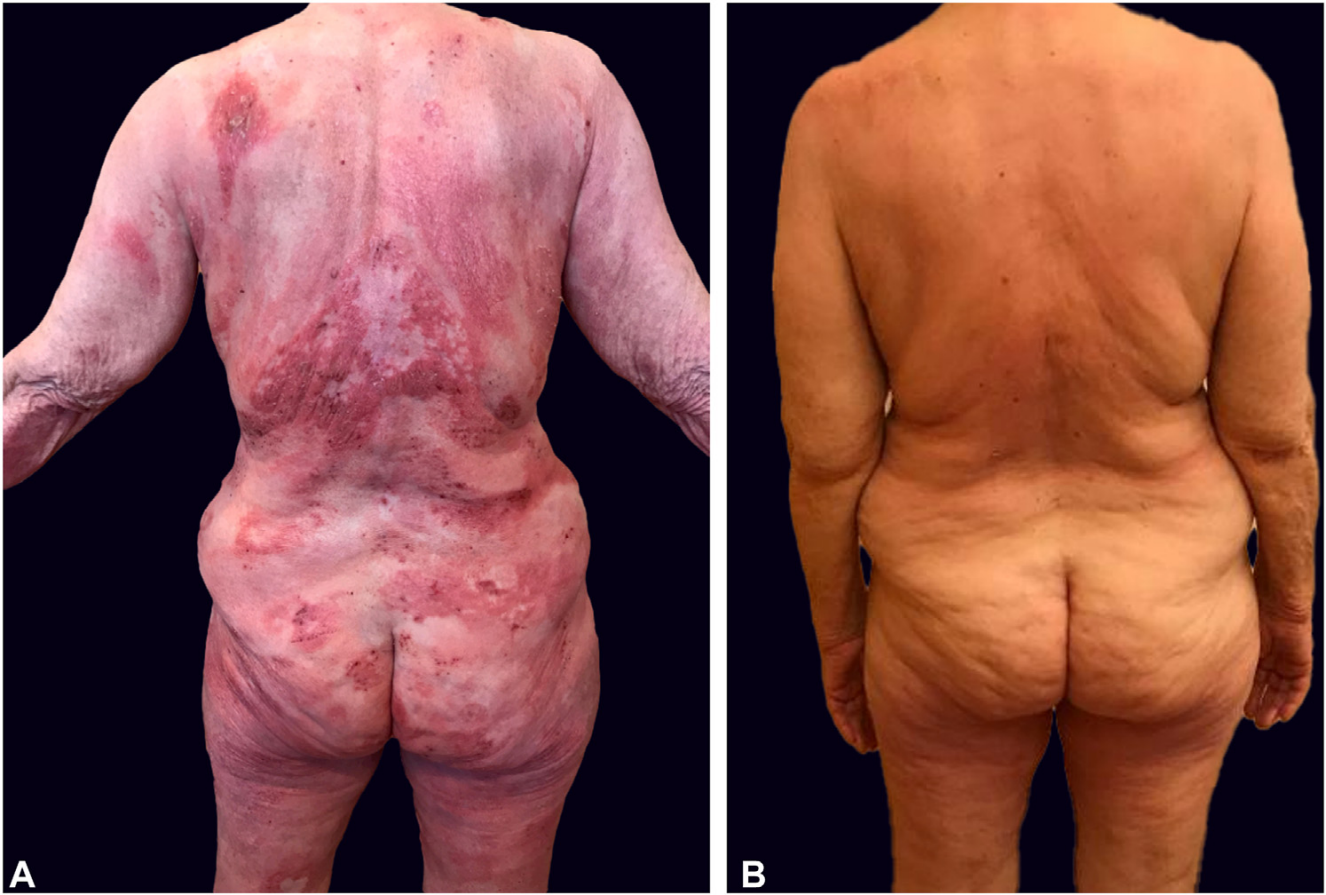
**A,** Skin deterioration with erythrodermic disease after initiation of Janus kinase (JAK)-inhibitor tofacitinib. **B,** Complete skin remission after 3 months of UVB-311nb phototherapy and acitretin.

The history of the disease coupled with the development of pronounced erythroderma and palmoplantar keratoderma following multiple cytokine-pathway blockers finally raised suspicion for CTCL (Fig 3, *A*). Subsequently, the patient was referred to a specialized cutaneous lymphoma clinic for diagnostic workup and follow-up treatment. While the psoriasiform histological pattern was retained in the follow-up skin biopsy, careful analysis by CTCL-expert dermatopathologists revealed atypical lymphocytic CD4+ infiltrate with epidermotropism and clonal TCR rearrangement as well (Fig 2, *C-H*). Moreover, a retrospective comparative clonality analysis confirmed the presence of an identical clone to the index clone in all preceding skin biopsies of the patient (*n* = 3). Clinical-pathological correlation along with PET-CT and immunophenotypic blood analysis negative for systemic involvement thus allowed the establishment, with a delay of 5 years from the initial symptoms, of the diagnosis of psoriasis-mimicking MF in advanced disease stage IIIA (T4N0M0B0). Three months upon initiation of MF stage-adapted treatment with UVB-311nb phototherapy and acitretin, a complete remission was reached (Fig 3, *B*).

## Discussion

This case raises a question regarding the impact of psoriasis medications on the progression of MF. The patient underwent various treatments since the initial presentation, but determining the precise influence of these interventions on MF progression is complex. While most of the therapies were administered to the patient before presenting at the specialized cutaneous lymphoma clinic, we had the opportunity to observe the clinical dynamics towards the end of the ixekizumab and during the tofacitinib treatment, when the first suspicions for MF started to appear. Although it is challenging to determine if any of the treatments negatively impacted the course of CTCL, our impression is that the disease accelerated during psoriasis-targeting approaches, diverging from a classical MF progression. We hypothesize that the rapid administration of various biologics targeting diverse pathways may have contributed to this acceleration. Additionally, we consider the withdrawal from these therapies as a factor favoring the observed control of MF in the patient. This assumption is supported by the relatively swift and excellent response of the advanced stage MF, with which the patient presented in our lymphoma clinic, to the combination of nbUVB and acitretin.^1^ This treatment combination is typically effective in early-stage disease but rarely in advanced-stage cases. Data considering the effect of cytokine-pathway blockers on the course of MF is scarce. In a recent multicenter study on 19 MF patients treated with biologics, therapy with IL-17A, -12/23 and -23 blockers has been associated with CTCL progression.^2^ Progression of undiagnosed MF under anti-IL17 treatment has been suggested to cause a shift in the regulatory T cells/Th17 balance towards regulatory T cells leading to further immunosuppression.^3^ In line with these findings, our patient was treated with IL23 blocker guselkumab and IL17A blocker ixekizumab with significant skin worsening. Activated JAK-signal transducer and activator of transcription signaling pathway plays a role in a number of inflammatory skin diseases. Consequently, use of JAK-inhibitors in dermatology is continuously increasing. These agents are currently employed in the management of a large variety of dermatologic entities, including common benign inflammatory dermatoses such as psoriasis, atopic dermatitis, or lichen planus.^4^ Deregulated JAK-signal transducer and activator of transcription pathway is also important for CTCL pathogenesis.^5^ Nevertheless, in a recent phase 2 biomarker-driven study of JAK 1/2 inhibitor ruxolitinib, in T-cell lymphomas, only 1 out of 7 patients with MF responded to this treatment.^6^ Our patient’s skin condition had significantly worsened after the initiation of JAK-inhibition. Data on the use of JAK-inhibitors in large CTCL cohorts is missing and their indication should thus be considered on a case-by-case basis in the absence of therapeutic alternatives. In the case of atypical dermatosis, we suggest that conventional systemic therapies (such as phototherapy and acitretin) might be preferable first-line choice compared to biologics. While a Th17 phenotype in MF is seldomly reported, it is not exceptional. It is of particular interest for a CTCL with a Th17 phenotype to worsen on anti-IL-17A therapy. This phenomenon is consistently observed in CTCL, particularly in its more classical manifestation: Th2-biased CTCL not responding or worsening under dupilumab. Thus, the delineation of CTCL from inflammatory skin diseases, especially atopic dermatitis and psoriasis, becomes a crucial clinical question in the modern era of targeting biological treatments. Multiple publications underline the importance of confirmatory skin biopsy prior to initiation of biologic therapy to exclude CTCL.^3^ In our patient, multiple biopsies revealed histological image of psoriasiform dermatitis. Moreover, we also observed a Th17-skewed inflammatory response in 3 independent skin biopsies by transcriptomic analysis. At the time of the first transcriptomic cytokine analysis, the patient was not receiving any treatment. Second biopsy was analyzed while the patient was on anti-IL17A treatment and third one was during therapy with JAK-inhibitor tofacitinib. Th17 phenotype remained stable during the disease course. Th17 phenotype of MF is rare as CTCL is more often characterized by a global Th2 bias of the T-cell repertoire^7^ with generally only a low level of Th17 cytokines. CTCL patients manifest with a high degree of intertumoral heterogeneity and show most commonly a distinct transcriptomic signature that seems to remain relatively stable during the disease course.^8^

Although psoriasis and MF might coexist,^9^ it does not seem to be the case in our patient as retrospective TCR clonality analysis revealed the presence of the same clone in all of the previous skin biopsies.

Two years after disease onset, our patient developed hair loss manifestations. Alopecia is reported in approximately 2.5% of CTCL patients with one-third of the cases developing alopecia areata-like patchy hair loss and two-thirds manifesting with alopecia within CTCL lesion.^10^ Alopecia areata has been recently reported as a comorbidity associated with MF, nevertheless the possibility of misdiagnosis of folliculotropic MF as alopecia areata has also been discussed.^11^ Although scalp lesions were not biopsied in our patient, the clinical findings in this context were consistent with alopecia areata-like lesions in the context of folliculotropic MF.

In summary, we report disease progression of MF misdiagnosed as psoriasis, and heavily pretreated with multiple cytokine blocking biologics. Atypical skin lesions can sometimes pose a diagnostic challenge, and assessment by dermatologists with specific expertise in MF can provide valuable insights. While psoriasiform dermatitis is common, the nuances that differentiate it from early-stage MF may require specialized knowledge. Given the potential for overlap and the significance of an accurate diagnosis, seeking the expertise of an MF specialist for the histopathology review can contribute to a more precise and reliable assessment.

## Conflicts of interest

None disclosed.
